# A LIFE COURSE PERSPECTIVE OF LATE-LIFE FUNCTIONING: THE STUDY OF WOMEN’S HEALTH ACROSS THE NATION

**DOI:** 10.1093/geroni/igz038.1984

**Published:** 2019-11-08

**Authors:** Carrie Karvonen-Gutierrez, Elsa Strotmeyer, Carol Derby

**Affiliations:** 1 University of Michigan, Ann Arbor, Michigan, United States; 2 University of Pittsburgh, Pittsburgh, Pennsylvania, United States; 3 Albert Einstein College of Medicine, Bronx, New York, United States

## Abstract

Early late-life deficits in physical functioning are highly relevant among women, who experience a more rapid decline in physical functioning vs. age-matched men. Trends showing an increasing prevalence of disability among mid-life adults, with an evolving understanding that late life health and functioning is the product of exposures accumulated across ones life, suggests an urgent need to understand the causes and consequences of functional limitations at the transition between mid- to late-life. The Study of Women’s Health Across the Nation (SWAN) is an observational multi-racial/ethnic (White, Black, Chinese, Japanese, Hispanic) longitudinal study of 3,302 women recruited in 1996 from 7 U.S. clinical sites; Boston, Chicago, Detroit-area, Los Angeles, Newark (NJ), Oakland (CA), and Pittsburgh. Over the past 23 years, women have participated in up to 16 near-annual study visits, spanning from mid-life (age 42-52 years at baseline) to late life (age 65-73 years at follow-up visit 15). Retention at the most recently completed in-person visit (2015) was 74%. This symposium identifies mid-life risk factors for poor late-life performance-based physical functioning among women, including race/ethnicity (Sternfeld) and chronic health conditions (Lange-Maia). Further, early late-life decrements in stair climb time and muscle power (Strotmeyer) and falls and fall injuries (Ylitalo) were observed with key factors identified. The Discussant will examine implications for life course epidemiology in advancing our understanding of predictors of late-life physical functioning. Consideration of functioning during the transition from mid-life to late life is critical to target interventions that are most efficacious in promoting late life health and function.

